# Can Cardiovascular Risk Factors Be Predicted? The Case of Weight‐Adjusted Waist Index

**DOI:** 10.1111/jch.70074

**Published:** 2025-05-23

**Authors:** Claudio Borghi, Giulia Fiorini

**Affiliations:** ^1^ Department of Cardiovascular Medicine IRCCS Azienda Ospedaliero‐Universitaria di Bologna University of Bologna Bologna Italy

1

In recent years, the quest for reliable indicators of metabolic health has intensified, particularly in the context of rising global obesity rates [1]. The increase in the prevalence of obesity is associated with an increase in cardiovascular and metabolic‐related mortality and morbidity that can be partially explained with the increase in the prevalence of several cardiometabolic risk factors, including hypertension, diabetes, lipid disorders, and elevated serum uric acid [2]. The traditional methods for assessing obesity, such as body mass index (BMI) and waist circumference, have their limitations since BMI does not account for fat distribution, while waist circumference fails to consider body weight. This problem has been recently emphasized by a publication of the Lancet Diabetes & Endocrinology Commission [3] suggesting the need for a new estimate of overweight/obesity to overcome the limitations of the current definitions. This is the reason why several alternative measures of overweight have come into play. Among the plethora of metrics available, the Weight‐Adjusted Waist Index (WWI) estimated by dividing the waist circumference (in cm) by the square root of body weight (in kg) [4] has emerged as a promising contender [5]. By adjusting waist circumference for weight, the WWI offers a more nuanced perspective on an individual's metabolic status. It effectively combines the advantages of both waist measurement and weight, providing a clearer picture of visceral fat accumulation, which is more closely associated with metabolic diseases.

In the present issue of the Journal, Miao et al. [6] have explored the potential of WWI as a predictive tool in patients with hypertension and hyperuricemia (HTN‐HUA), two conditions that are increasingly prevalent and often interlinked. Hypertension is a major risk factor for cardiovascular diseases and is often exacerbated by obesity [7]. Hyperuricemia, characterized by elevated uric acid levels in the blood, is not only a precursor to gout but is also associated with various metabolic disorders, including hypertension [8]. Hypertension and elevated urate levels are frequently associated in the same patients, particularly in the presence of obesity [9, 10] that could represent an early promoter of both cardiometabolic risk factors. The interplay between these three conditions underscores the need for effective screening tools that can identify individuals at risk before the onset of more severe health issues.

The study has examined the population of the National Health and Nutrition Examination Survey (NHANES) database during the period 1999–2018 and has reached the conclusion of a significant and nonlinear association between WWI and HTN‐HUA. A weaker, but significant correlation has also been observed between WWI and SUA or HTN alone, suggesting the predictive role of such a novel index of overweight in the identification of patients with multiple cardiometabolic risk factors.

The results agree with emerging studies suggest that WAWI may be a strong predictor of HTN‐HUA [11]. Increased blood pressure and serum uric acid are closely correlated, and several papers in the past years [8, 12] have demonstrated that elevated SUA can precede the increase in blood pressure values according to a mechanistic relationship mainly based on the level of oxidative stress produced by the activity of xanthine‐oxidase, that is, the primary pathway to produce circulating uric acid [13]. The rationale behind the evidence provided by the paper of Miao et al. [6] is straightforward: a higher WWI indicates a greater proportion of abdominal fat relative to body weight, which is a significant risk factor for both conditions. The best support to this interpretation is probably the metabolic syndrome, which often combines the presence of overweight with high blood pressure and increased SUA levels, resulting in an excess in the risk of cardiovascular disease [14]. In particular, overweight, hypertension, and elevated SUA contribute to reduce insulin sensitivity that is largely known as a common promoter of cardiometabolic mortality and morbidity. As visceral fat is known to produce inflammatory markers and disrupt metabolic regulation, it becomes imperative to monitor this index, particularly in populations with high obesity rates. The evaluation of WWI might represent a simple approach to identify the presence of an underlying condition of insulin resistance and could be adopted for the extensive screening of the general population with the scope of an early prevention of cardiometabolic diseases. Interestingly, the results of the paper of Miao et al. [6] suggest that the predictive role of WWI on HTN‐SUA condition is more evident in younger subjects and in patients with normal body weight, where the future onset of cardiometabolic diseases is usually hard to predict. This observation has remarkable practical implications since the measure of WWi can be easily achieved and could be introduced to improve the preventive policies in to the general population of the world. Furthermore, the implementation of WWI in clinical practice could enhance our ability to stratify risk among subjects with mild to moderate risk of cardiometabolic disease and contribute to the reclassification of subjects amenable of a more aggressive preventive strategy. For healthcare providers, incorporating WWI into routine assessments could facilitate early intervention strategies, such as lifestyle modifications or pharmacotherapy, aimed at reducing hypertension and managing uric acid levels. This proactive approach could lead to improve patient outcomes and potentially reduce the burden on healthcare systems.

However, while the initial findings surrounding WWI are promising, further research is necessary to validate its efficacy across diverse populations and age groups. Longitudinal studies that track the relationship between WAWI and the onset of HTN‐HUA will be crucial in establishing its utility as a standard clinical tool. Additionally, understanding the mechanisms underlying the association between WWI and these conditions will inform targeted prevention strategies.

In the paper of Miao et al. [6], the major limitation of the use of WWI as a predictive tool, is the fact that the information that can be drawn is limited to only two of the components of the composite cardiometabolic risk profile. No evidence is provided about the relationship between WWI and glucose control or lipid profile, considered the major determinants of future cardiometabolic profile Included a strong impact on the development of high blood pressure and uric acid disorders. This drawback can be partially limited by statistical adjustments, whose impact is, however, significantly reduced by the confounding effects of collinearity and pathophysiological interactions among risk factors. An additional limitation of the study is the definition of hyperuricemia, that is, based on a “rheumatological” approach that identifies abnormal serum uric acid levels based on the risk of developing gout. Conversely, many evidence has been published supporting a lower threshold of serum urate for the development of cardiovascular disease, including myocardial infarction, stroke, and heart failure [15, 16, 17]. Virdis et al. [15] have reported a significant increase in the relative risk of cardiovascular disease in subjects with serum uric acid levels between 4.5 and 5.5 mg/dL. Similar results have been provided in the population of the Rotterdam study [16] and in a large Chinese population where the cardiovascular impact of elevated uric acid has been evaluated in subjects without additional risk factors for cardiovascular disease [17]. A lower threshold for the definition of hyperuricemia might increase the predictive role of the WWI with the inclusion of a proportion of subjects at risk of cardiometabolic disease and currently included in the normal subgroup and, for this reason, excluded from the preventive strategies. This broader approach would not produce an increase in the resources required for the measure and interpretation of the potential role of WWI with a potential advantage in terms of a number of subjects that could benefit of an early approach to cardiometabolic prevention.

In conclusion, the WWI presents a compelling case as a predictive measure for HTN‐HUA. As we strive to address the growing epidemic of metabolic disorders, it is vital that we embrace innovative tools that provide deeper insights into our patients’ health. The adoption of WAWI could represent a significant step forward in our efforts to combat these interconnected health challenges, ultimately paving the way for a healthier future.
